# (*Z*)-3-(3-Phenyl­allyl­idene)-1,5-dioxa­spiro­[5.5]undecane-2,4-dione

**DOI:** 10.1107/S1600536809038781

**Published:** 2009-09-30

**Authors:** Wu-Lan Zeng, Hua-Xiang Zhang, Fang-Fang Jian

**Affiliations:** a MicroScale Science Institute, Department of Chemistry and Chemical Engineering, Weifang University, Weifang 261061, People’s Republic of China; b MicroScale Science Institute, Weifang University, Weifang 261061, People’s Republic of China

## Abstract

In the title compound, C_18_H_18_O_4_, the 1,3-dioxane ring adopts a distorted envelope conformation with the C atom common to the cyclo­hexane ring forming the flap. In the crystal, inversion dimers linked by pairs of C—H⋯O hydrogen bonds occur.

## Related literature

For background information on spiro-compounds, see: Jiang *et al.* (1998 ▶); Lian *et al.* (2008 ▶); Wei *et al.* (2008 ▶).
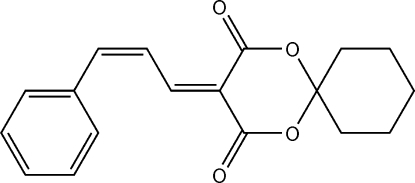


## Experimental

### 

#### Crystal data

C_18_H_18_O_4_
*M* *_r_* = 298.32
Triclinic, 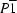

*a* = 7.1177 (14) Å
*b* = 9.5506 (19) Å
*c* = 11.734 (2) Å
α = 106.82 (3)°
β = 100.14 (3)°
γ = 93.35 (3)°
*V* = 746.6 (3) Å^3^
*Z* = 2
Mo *K*α radiation
μ = 0.09 mm^−1^
*T* = 293 K
0.22 × 0.18 × 0.10 mm

#### Data collection

Bruker SMART CCD diffractometer
Absorption correction: none
7448 measured reflections
3401 independent reflections
2309 reflections with *I* > 2σ(*I*)
*R*_int_ = 0.016

#### Refinement

*R*[*F* ^2^ > 2σ(*F* ^2^)] = 0.034
*wR*(*F* ^2^) = 0.130
*S* = 1.17
3401 reflections
199 parameters
H-atom parameters constrained
Δρ_max_ = 0.30 e Å^−3^
Δρ_min_ = −0.21 e Å^−3^

### 

Data collection: *SMART* (Bruker, 1997 ▶); cell refinement: *SAINT* (Bruker, 1997 ▶); data reduction: *SAINT*; program(s) used to solve structure: *SHELXS97* (Sheldrick, 2008 ▶); program(s) used to refine structure: *SHELXL97* (Sheldrick, 2008 ▶); molecular graphics: *SHELXTL* (Sheldrick, 2008 ▶); software used to prepare material for publication: *SHELXTL*.

## Supplementary Material

Crystal structure: contains datablocks global, I. DOI: 10.1107/S1600536809038781/hb5108sup1.cif

Structure factors: contains datablocks I. DOI: 10.1107/S1600536809038781/hb5108Isup2.hkl

Additional supplementary materials:  crystallographic information; 3D view; checkCIF report

## Figures and Tables

**Table 1:** Hydrogen-bond geometry (Å, °)

*D*—H⋯*A*	*D*—H	H⋯*A*	*D*⋯*A*	*D*—H⋯*A*
C10—H10*A*⋯O2^i^	0.97	2.52	3.440 (2)	158

Symmetry code: (i) 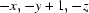.

**Fig. 1.**
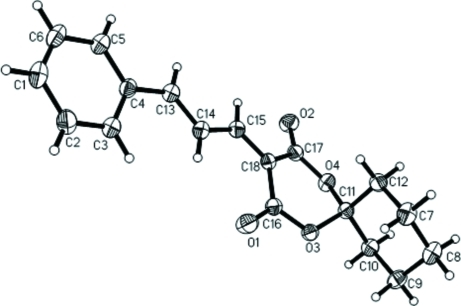
The molecular structure of (I), drawn with 30% probability ellipsoids and spheres of arbritrary size for the H atoms.

## supplementary crystallographic information

### Comment

Spiro compounds are widely used in medicine, catalysis and optical materials (Lian *et al.*, 2008; Jiang *et al.*, 1998; Wei *et al.*, 2008) owing to their interesting conformational features. We report here the synthesis and structure of the title compound, (I) (Fig. 1), as part of our ongoing studies on new spiro compounds with potentially higher bioactivity.

The 1,3-dioxane ring is in a distored envelope conformation with atom C11 atom common to the cyclohexane forming the flap. The crystal structure is stabilized by weak intermolecular C—H···O hydrogen bonds (Table 1).

### Experimental

A mixture of malonic acid (6.24 g, 0.06 mol) and acetic anhydride(9 ml) in strong sulfuric acid (0.25 ml) was stirred with water at 303K, After dissolving, cyclohexanone (5.88 g, 0.06 mol) was added dropwise into solution for 1 h. The reaction was allowed to proceed for 4 h. The mixture was cooled and filtered, and then an ethanol solution of (Z)-3-phenylacrylaldehyde (7.92g, 0.06 mol) was added. The solution was then filtered and concentrated.  Yellow blocks of (I) were obtained by evaporation of a petroleum ether–ethylacetate (3:1 *v*/*v*) solution at room temperature over a period of one week.

### Refinement

The H atoms were placed in calculated positions (C—H = 0.93–0.97 Å), and refined as riding with *U*_iso_(H) = 1.2*U*_eq_(C).

### Crystal data

**Table:**

C_18_H_18_O_4_	*Z* = 2
*M**_r_* = 298.32	*F*(000) = 316
Triclinic, *P*1	*D*_x_ = 1.327 Mg m^−^^3^
Hall symbol: -P 1	Mo *K*α radiation, λ = 0.71073 Å
*a* = 7.1177 (14) Å	Cell parameters from 3401 reflections
*b* = 9.5506 (19) Å	θ = 3.1–27.5°
*c* = 11.734 (2) Å	µ = 0.09 mm^−^^1^
α = 106.82 (3)°	*T* = 293 K
β = 100.14 (3)°	Block, yellow
γ = 93.35 (3)°	0.22 × 0.18 × 0.10 mm
*V* = 746.6 (3) Å^3^	

### Data collection

**Table:**

Bruker SMART CCD diffractometer	2309 reflections with *I* > 2σ(*I*)
Radiation source: fine-focus sealed tube	*R*_int_ = 0.016
graphite	θ_max_ = 27.5°, θ_min_ = 3.1°
ω scans	*h* = −8→9
7448 measured reflections	*k* = −12→12
3401 independent reflections	*l* = −15→15

### Refinement

**Table:**

Refinement on *F*^2^	Primary atom site location: structure-invariant direct methods
Least-squares matrix: full	Secondary atom site location: difference Fourier map
*R*[*F*^2^ > 2σ(*F*^2^)] = 0.034	Hydrogen site location: inferred from neighbouring sites
*wR*(*F*^2^) = 0.130	H-atom parameters constrained
*S* = 1.17	*w* = 1/[σ^2^(*F*_o_^2^) + (0.0724*P*)^2^] where *P* = (*F*_o_^2^ + 2*F*_c_^2^)/3
3401 reflections	(Δ/σ)_max_ < 0.001
199 parameters	Δρ_max_ = 0.30 e Å^−^^3^
0 restraints	Δρ_min_ = −0.21 e Å^−^^3^

### Special details

**Table:**

Geometry. All e.s.d.'s (except the e.s.d. in the dihedral angle between two l.s. planes) are estimated using the full covariance matrix. The cell e.s.d.'s are taken into account individually in the estimation of e.s.d.'s in distances, angles and torsion angles; correlations between e.s.d.'s in cell parameters are only used when they are defined by crystal symmetry. An approximate (isotropic) treatment of cell e.s.d.'s is used for estimating e.s.d.'s involving l.s. planes.
Refinement. Refinement of *F*^2^ against ALL reflections. The weighted *R*-factor *wR* and goodness of fit *S* are based on *F*^2^, conventional *R*-factors *R* are based on *F*, with *F* set to zero for negative *F*^2^. The threshold expression of *F*^2^ > σ(*F*^2^) is used only for calculating *R*-factors(gt) *etc*. and is not relevant to the choice of reflections for refinement. *R*-factors based on *F*^2^ are statistically about twice as large as those based on *F*, and *R*- factors based on ALL data will be even larger.

### Fractional atomic coordinates and isotropic or equivalent isotropic displacement parameters (Å^2^)

**Table:**

	*x*	*y*	*z*	*U*_iso_*/*U*_eq_	
O4	0.07551 (13)	0.64682 (9)	0.13593 (9)	0.0440 (3)	
O3	0.27862 (12)	0.86963 (9)	0.18966 (9)	0.0435 (3)	
C18	−0.01600 (18)	0.86915 (14)	0.26210 (12)	0.0386 (3)	
O2	−0.18747 (14)	0.63080 (11)	0.20875 (11)	0.0555 (3)	
C17	−0.05451 (19)	0.70896 (14)	0.20042 (13)	0.0408 (3)	
C11	0.19207 (18)	0.73899 (13)	0.09196 (12)	0.0375 (3)	
C12	0.07076 (19)	0.77924 (15)	−0.01133 (13)	0.0443 (3)	
H12A	−0.0251	0.8396	0.0195	0.053*	
H12B	0.0042	0.6902	−0.0723	0.053*	
C10	0.35558 (19)	0.65608 (14)	0.05319 (14)	0.0440 (3)	
H10A	0.3043	0.5604	−0.0042	0.053*	
H10B	0.4353	0.6405	0.1235	0.053*	
C16	0.16915 (19)	0.94609 (15)	0.26336 (13)	0.0443 (3)	
C4	−0.2995 (2)	1.28109 (15)	0.52506 (13)	0.0430 (3)	
C14	−0.1418 (2)	1.08555 (15)	0.39392 (13)	0.0450 (3)	
H14A	−0.0315	1.1500	0.4069	0.054*	
C15	−0.15033 (19)	0.93622 (15)	0.32054 (13)	0.0426 (3)	
H15A	−0.2635	0.8767	0.3116	0.051*	
O1	0.23340 (15)	1.06908 (12)	0.32633 (13)	0.0728 (4)	
C13	−0.2902 (2)	1.13463 (16)	0.44451 (13)	0.0453 (3)	
H13A	−0.3994	1.0674	0.4260	0.054*	
C5	−0.4695 (2)	1.31394 (17)	0.56535 (14)	0.0511 (4)	
H5A	−0.5745	1.2418	0.5408	0.061*	
C9	0.4779 (2)	0.74002 (16)	−0.00542 (15)	0.0520 (4)	
H9A	0.5422	0.8302	0.0551	0.062*	
H9B	0.5757	0.6807	−0.0346	0.062*	
C3	−0.1442 (2)	1.39115 (17)	0.56447 (14)	0.0505 (4)	
H3A	−0.0287	1.3716	0.5399	0.061*	
C6	−0.4845 (2)	1.45175 (18)	0.64115 (15)	0.0576 (4)	
H6A	−0.5987	1.4717	0.6676	0.069*	
C8	0.3571 (2)	0.77756 (18)	−0.11085 (16)	0.0584 (4)	
H8A	0.3042	0.6875	−0.1755	0.070*	
H8B	0.4376	0.8363	−0.1426	0.070*	
C2	−0.1606 (2)	1.52867 (18)	0.63955 (15)	0.0585 (4)	
H2A	−0.0563	1.6015	0.6649	0.070*	
C7	0.1938 (2)	0.86284 (17)	−0.06957 (15)	0.0551 (4)	
H7A	0.2471	0.9577	−0.0115	0.066*	
H7B	0.1140	0.8803	−0.1390	0.066*	
C1	−0.3313 (3)	1.55903 (18)	0.67735 (15)	0.0590 (4)	
H1A	−0.3420	1.6523	0.7274	0.071*	

### Atomic displacement parameters (Å^2^)

**Table:**

	*U*^11^	*U*^22^	*U*^33^	*U*^12^	*U*^13^	*U*^23^
O4	0.0515 (5)	0.0329 (5)	0.0487 (6)	0.0045 (4)	0.0164 (4)	0.0102 (4)
O3	0.0393 (5)	0.0384 (5)	0.0451 (6)	0.0004 (4)	0.0101 (4)	0.0006 (4)
C18	0.0396 (6)	0.0387 (7)	0.0341 (7)	0.0034 (5)	0.0056 (5)	0.0072 (6)
O2	0.0517 (6)	0.0479 (6)	0.0653 (8)	−0.0055 (5)	0.0172 (5)	0.0136 (5)
C17	0.0412 (7)	0.0411 (7)	0.0386 (7)	0.0026 (6)	0.0063 (6)	0.0112 (6)
C11	0.0407 (6)	0.0300 (6)	0.0389 (7)	0.0024 (5)	0.0092 (5)	0.0059 (5)
C12	0.0454 (7)	0.0429 (7)	0.0438 (8)	0.0112 (6)	0.0078 (6)	0.0113 (6)
C10	0.0470 (7)	0.0359 (6)	0.0493 (8)	0.0123 (6)	0.0113 (6)	0.0110 (6)
C16	0.0415 (7)	0.0416 (7)	0.0428 (8)	0.0034 (6)	0.0089 (6)	0.0024 (6)
C4	0.0470 (7)	0.0489 (7)	0.0366 (7)	0.0114 (6)	0.0134 (6)	0.0144 (6)
C14	0.0458 (7)	0.0465 (7)	0.0407 (8)	0.0060 (6)	0.0115 (6)	0.0082 (6)
C15	0.0420 (7)	0.0462 (7)	0.0384 (7)	0.0034 (6)	0.0086 (6)	0.0112 (6)
O1	0.0542 (6)	0.0493 (6)	0.0873 (9)	−0.0122 (5)	0.0238 (6)	−0.0238 (6)
C13	0.0448 (7)	0.0481 (7)	0.0426 (8)	0.0063 (6)	0.0109 (6)	0.0118 (6)
C5	0.0522 (8)	0.0555 (8)	0.0493 (9)	0.0097 (7)	0.0199 (7)	0.0151 (7)
C9	0.0492 (8)	0.0500 (8)	0.0612 (10)	0.0158 (7)	0.0235 (7)	0.0145 (7)
C3	0.0492 (8)	0.0571 (9)	0.0440 (9)	0.0071 (7)	0.0147 (6)	0.0104 (7)
C6	0.0644 (10)	0.0632 (9)	0.0533 (10)	0.0229 (8)	0.0298 (8)	0.0165 (8)
C8	0.0696 (10)	0.0579 (9)	0.0577 (10)	0.0154 (8)	0.0298 (8)	0.0216 (8)
C2	0.0685 (10)	0.0551 (9)	0.0474 (9)	0.0000 (8)	0.0143 (8)	0.0086 (8)
C7	0.0685 (10)	0.0530 (8)	0.0541 (10)	0.0214 (7)	0.0191 (8)	0.0253 (8)
C1	0.0802 (11)	0.0530 (9)	0.0458 (9)	0.0170 (8)	0.0236 (8)	0.0099 (7)

### Geometric parameters (Å, °)

**Table:**

O4—C17	1.3536 (17)	C14—C15	1.428 (2)
O4—C11	1.4344 (16)	C14—H14A	0.9300
O3—C16	1.3515 (17)	C15—H15A	0.9300
O3—C11	1.4437 (16)	C13—H13A	0.9300
C18—C15	1.3575 (19)	C5—C6	1.381 (2)
C18—C16	1.4665 (19)	C5—H5A	0.9300
C18—C17	1.4765 (19)	C9—C8	1.522 (2)
O2—C17	1.2062 (17)	C9—H9A	0.9700
C11—C10	1.5080 (18)	C9—H9B	0.9700
C11—C12	1.5174 (18)	C3—C2	1.378 (2)
C12—C7	1.522 (2)	C3—H3A	0.9300
C12—H12A	0.9700	C6—C1	1.369 (2)
C12—H12B	0.9700	C6—H6A	0.9300
C10—C9	1.524 (2)	C8—C7	1.526 (2)
C10—H10A	0.9700	C8—H8A	0.9700
C10—H10B	0.9700	C8—H8B	0.9700
C16—O1	1.2045 (17)	C2—C1	1.384 (2)
C4—C3	1.394 (2)	C2—H2A	0.9300
C4—C5	1.395 (2)	C7—H7A	0.9700
C4—C13	1.457 (2)	C7—H7B	0.9700
C14—C13	1.344 (2)	C1—H1A	0.9300
			
C17—O4—C11	118.14 (10)	C14—C15—H15A	115.5
C16—O3—C11	119.54 (10)	C14—C13—C4	127.39 (14)
C15—C18—C16	123.28 (12)	C14—C13—H13A	116.3
C15—C18—C17	117.99 (12)	C4—C13—H13A	116.3
C16—C18—C17	118.63 (12)	C6—C5—C4	121.11 (15)
O2—C17—O4	118.86 (12)	C6—C5—H5A	119.4
O2—C17—C18	124.36 (14)	C4—C5—H5A	119.4
O4—C17—C18	116.68 (12)	C8—C9—C10	111.66 (12)
O4—C11—O3	110.01 (11)	C8—C9—H9A	109.3
O4—C11—C10	107.54 (10)	C10—C9—H9A	109.3
O3—C11—C10	106.20 (10)	C8—C9—H9B	109.3
O4—C11—C12	109.82 (10)	C10—C9—H9B	109.3
O3—C11—C12	110.77 (10)	H9A—C9—H9B	107.9
C10—C11—C12	112.40 (12)	C2—C3—C4	120.55 (15)
C11—C12—C7	111.29 (12)	C2—C3—H3A	119.7
C11—C12—H12A	109.4	C4—C3—H3A	119.7
C7—C12—H12A	109.4	C1—C6—C5	119.95 (15)
C11—C12—H12B	109.4	C1—C6—H6A	120.0
C7—C12—H12B	109.4	C5—C6—H6A	120.0
H12A—C12—H12B	108.0	C9—C8—C7	110.64 (13)
C11—C10—C9	111.33 (10)	C9—C8—H8A	109.5
C11—C10—H10A	109.4	C7—C8—H8A	109.5
C9—C10—H10A	109.4	C9—C8—H8B	109.5
C11—C10—H10B	109.4	C7—C8—H8B	109.5
C9—C10—H10B	109.4	H8A—C8—H8B	108.1
H10A—C10—H10B	108.0	C3—C2—C1	120.34 (16)
O1—C16—O3	117.67 (13)	C3—C2—H2A	119.8
O1—C16—C18	125.75 (14)	C1—C2—H2A	119.8
O3—C16—C18	116.55 (12)	C12—C7—C8	111.50 (12)
C3—C4—C5	118.04 (14)	C12—C7—H7A	109.3
C3—C4—C13	122.64 (13)	C8—C7—H7A	109.3
C5—C4—C13	119.32 (14)	C12—C7—H7B	109.3
C13—C14—C15	121.18 (14)	C8—C7—H7B	109.3
C13—C14—H14A	119.4	H7A—C7—H7B	108.0
C15—C14—H14A	119.4	C6—C1—C2	120.00 (16)
C18—C15—C14	128.93 (13)	C6—C1—H1A	120.0
C18—C15—H15A	115.5	C2—C1—H1A	120.0

### Hydrogen-bond geometry (Å, °)

**Table:**

*D*—H···*A*	*D*—H	H···*A*	*D*···*A*	*D*—H···*A*
C10—H10A···O2^i^	0.97	2.52	3.440 (2)	158

Symmetry codes: (i) −*x*, −*y*+1, −*z*.
